# A Single Hot Event That Does Not Affect Survival but Decreases Reproduction in the Diamondback Moth, *Plutella xylostella*

**DOI:** 10.1371/journal.pone.0075923

**Published:** 2013-10-08

**Authors:** Wei Zhang, Fei Zhao, Ary A. Hoffmann, Chun-Sen Ma

**Affiliations:** 1 Climate Change Biology Research Group, State Key Laboratory for Biology of Plant Diseases and Insect Pests, Institute of Plant Protection, Chinese Academy of Agricultural Sciences, Beijing, China; 2 Departments of Zoology and Genetics, Bio21 Institute, The University of Melbourne, Victoria, Australia; USDA-Agricultural Research Service, United States of America

## Abstract

Extremely hot events (usually involving a few hours at extreme high temperatures in summer) are expected to increase in frequency in temperate regions under global warming. The impact of these events is generally overlooked in insect population prediction, since they are unlikely to cause widespread mortality, however reproduction may be affected by them. In this study, we examined such stress effects in the diamondback moth, *Plutella xylostella*. We simulated a single extreme hot day (maximum of 40°C lasting for 3, 4 or 5 h) increasingly experienced under field conditions. This event had no detrimental effects on immediate mortality, copulation duration, mating success, longevity or lifetime fecundity, but stressed females produced 21% (after 3 or 4 h) fewer hatched eggs because of a decline in the number and hatching success of eggs laid on the first two days. These negative effects on reproduction were no longer evident in the following days. Male heat exposure led to a similar but smaller effect on fertile egg production, and exposure extended pre-mating period in both sexes. Our results indicate that a single hot day can have detrimental effects on reproduction, particularly through maternal effects on egg hatching, and thereby influence the population dynamics of diamondback moth.

## Introduction

**Figure 1:**
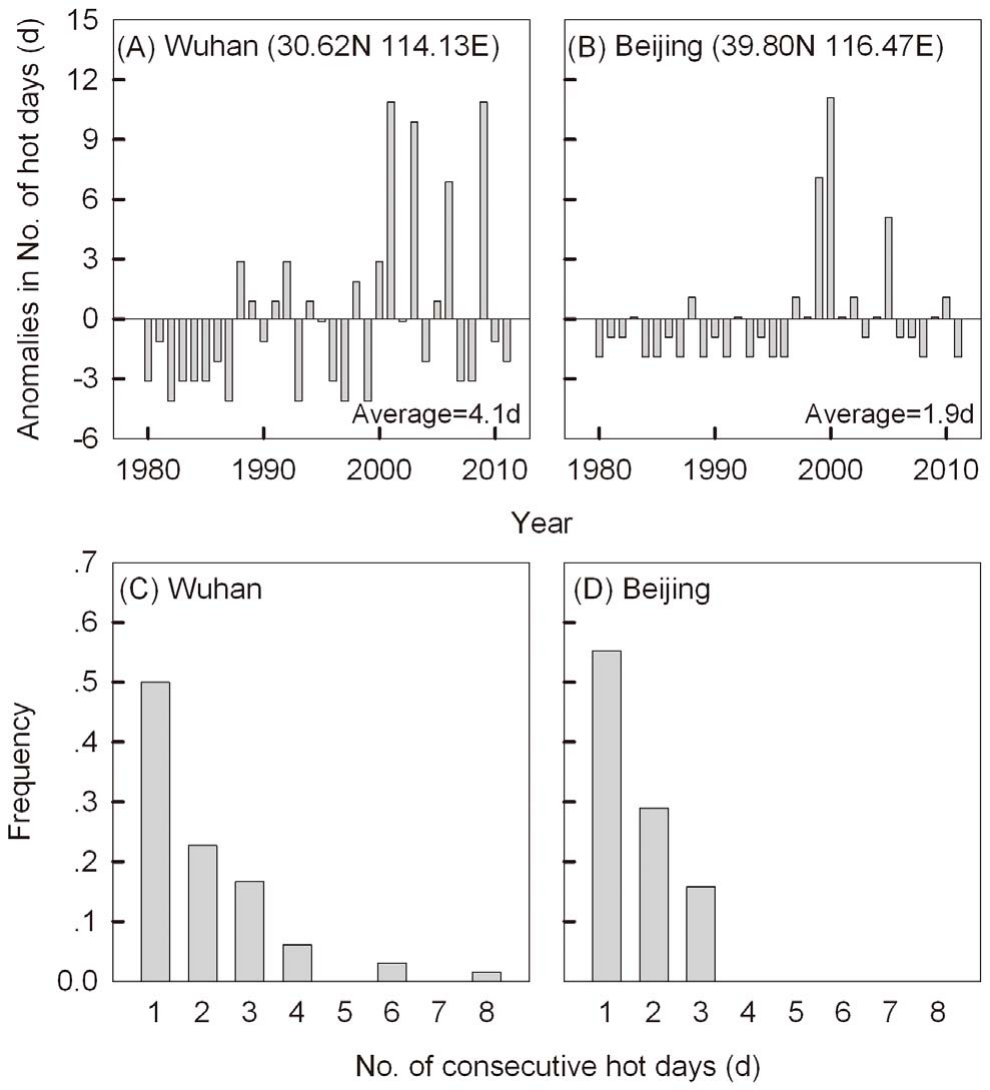
Hot days (DTmax ≥37°C) in June-August at Wuhan and Beijing from 1980–2011. Anomalies in the number of hot days (to the average form 1980–2011) at Wuhan (A) and Beijing (B) and the frequency of the consecutive hot days at Wuhan (C) and Beijing (D). Daily maximum temperature records were downloaded from the China Meteorological Data Sharing Service System. High temperatures 10 cm above ground and shadowed with Brassica leaves in fields recorded with data-loggers are normally 3°C higher than temperatures recorded at weather stations at Wuhan.

Ambient temperatures often fluctuate with occasional periods of extreme high temperatures. These periods are predicted to become more intense and occur more often in the future [1]–[4] in many areas of agricultural production around the world. For instance, from June to August (the crop growing season) near Beijing and Wuhan in China, daily maximum ambient temperatures (DT_max_) often exceed 37°C and the number of hot days when this temperature is reached appears to be increasing in the last 10 years (Fig. 1, A and B). These conditions translate into exposures of >40°C for insects like the diamondback moth, *Plutella xylostella*, which is a pest living among foliage in *Brassica* field crops where temperatures are around 3°C higher than ambient conditions (Ma, unpublished data).

High temperature affects the population performance of insects under field conditions [5]–[7]. Temperature effects are typically studied by exposing organisms either to constant high temperatures or repeated heat stress. Exposing insects to constant high temperature for an entire generation or across multiple generations often leads to a population collapse [8]–[10]. Maintaining insects at high temperatures for a few days can also slow population growth by direct mortality and reproduction inhibition [11], [12], while repeated heat stress has been shown to affect the lifetime fecundity and longevity of *Metopolophium* [13], and the fecundity and longevity of *Drosophila* [14]–[16].

However, high temperatures under field conditions might only occur on a single day and last for a few hours (Fig. 1, C and D), having little impact on the mean temperature experienced across a generation. Since insects can usually become hardened [17], [18], occasional high temperature events do not necessarily result in mortality and they have therefore rarely been considered in population prediction. Nevertheless, single events might influence reproductive traits that are vulnerable to high temperature [19]–[21] and offspring might also be affected by high maternal temperatures [22]–[24]. Hence, high thermal sensitivity of egg production and egg hatching raise the issue of whether a single hot event in agricultural areas might influence the performance of pests and perhaps influence damage levels.

Here we address the effects of a single extreme event on the diamondback moth (DBM, *P. xylostella*), the most destructive pest of cruciferous crops around the world. The species is well known for its ability to persist in stressed environments, such as under cold conditions (reviewed in [25], [26]) and in the presence of insecticides [27], [28], although it does not undergo diapause [29]. Detailed information is available about the effects of constant temperatures on development, survival and reproduction of this species [30]–[36].

We focus on the effects of a single extreme event at the adult stage. DBM is uncommon in fields at Wuhan in summer even though mean temperatures during the hottest periods (for instance, 29.5°C, July 15^th^ to August 15^th^ during 1980–2011) are lower than highest temperatures at which DBM can be cultured (32°C recorded in [31]). We therefore considered the following questions. 1) Can a single hot event adversely affect reproduction and consequently the population dynamics of DBM? 2) If this is the case, which fitness component and parental sex is relatively more vulnerable? To answer these questions, we investigated the effects of a single stress at 40°C for 0, 3, 4 or 5 h exposure on different aspects of adult performance: mating behavior, longevity, fecundity and egg hatching rate through parental effects.

## Materials and Methods

### Stock Rearing

The population of DBM larvae was collected from *Brassica* fields in May 2010, with the permission of the Experiment Station of Hubei Academy of Agricultural Sciences, in Wuhan, Hubei Province, China. Larvae were reared on an artificial diet (Southland Products Incorporated, USA) in large plastic boxes (10×10×9 cm) placed at 25±1°C, 30–40% RH and 15L: 9D. Scotophase was set between 20∶00 to 05∶00. Pupae were moved to screen cages (30×25×30 cm) for adult eclosion.

### Test Insects

Nearly 800 pairs of adults were fed by small cotton balls dipped with 5% honey solution, and allowed to mate in screen cages. Two days after eclosion, egg cards (5×10 cm) made of Parafilm (American Can Co.) were dipped in cabbage juice and hung on the top of cages 1 h before lights were switched off, and left for 12 h to collect the eggs. Egg cards were renewed the next day following the same procedure as on the first day. Cards with nearly 500 fresh eggs were hung over the larval rearing box for hatching (110 g diet/box). The newly hatched larvae fell down and then fed on the artificial diet to develop from 1st to 4th-instar, and then pupated. From the F1s, 500 pupae for the first 2 days were selected and separated singly in glass tubes (10 cm long and 1.5 cm diameter). DBM eclosion was checked every day and adults sexed.

Emerging 3-day-old females and males were used in our experiments. Finally, 89, 86 and 77 females and males exposed to 40°C for 3, 4 or 5 h respectively. We supplied 5% honey solution 2 h before heat treatment.

### Experimental Design

Females and males were randomly divided among two treatment groups: one remained in the climate room at 25°C (control), while the other was placed at 40°C (heat stress, 60–70% RH) for 3, 4 or 5 h. To analyze heat effects on mating behavior, fecundity and fertility, individuals were mated in all four possible sex by treatment combinations: control females and males (25/25), control females and stressed males (25/40), stressed females and control males (40/25) and stressed females and stressed males (40/40).

### Mating

**Figure 2:**
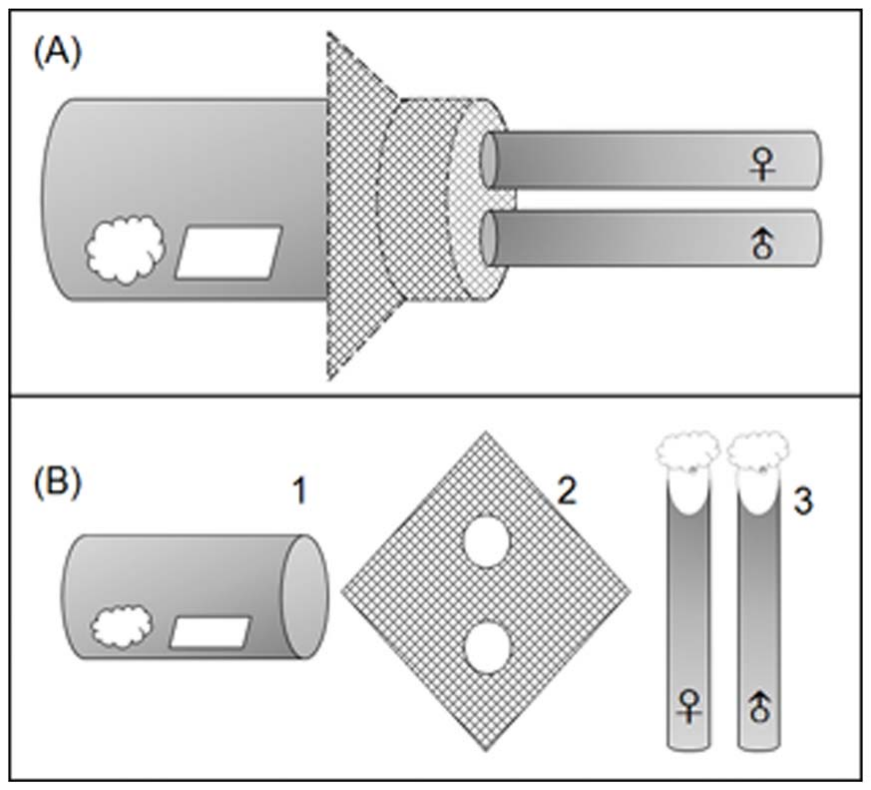
The experimental device for DBM mating operation (A) and its components (B). The device as depicted in (B) includes (1) a mating glass in which an egg card and a cotton ball with 5% honey solution were provided for egg laying and feeding; (2) a nylon net with two holes, allowing females and males in the tubes walking freely to the mating glass; and (3) two tubes for collecting females and males separately.

**Figure 3:**
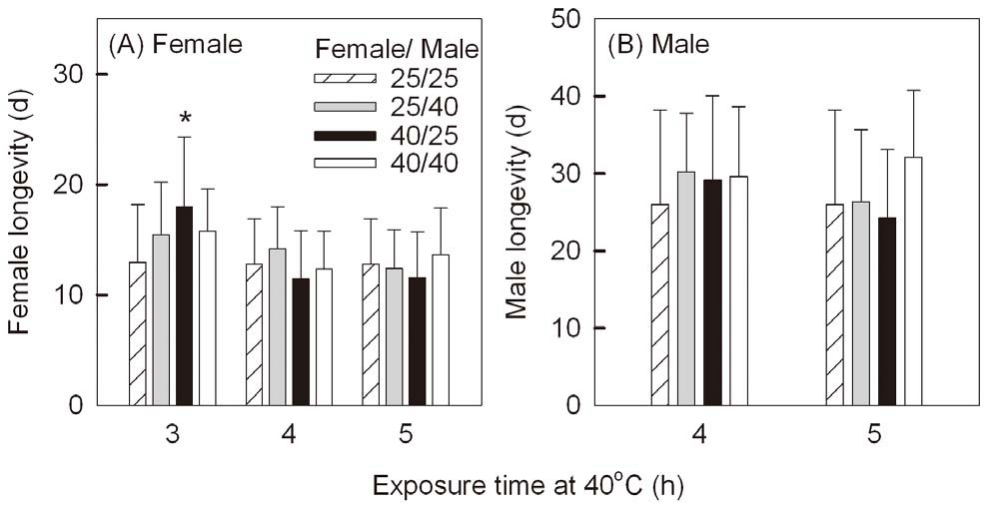
Mean longevity (±SD) after different treatments. (A) and (B) show females and males respectively. Treatments involved combinations of females and males exposed to control (25°C) or stress (40°C) treatments for 3, 4 or 5 h. In the control group, both sexes were held at 25°C. “*”indicate significant differences (P<0.05) from the controls (both sexes at 25°C).

DBM adults display end-to-end mating and successful copulation typically lasts over 30 min [37], [38]. Thus end-to-end copula over 30 min was used as a criterion for successful mating. In the experiments, males and females were separated in two tubes (Fig. 2, B-3). The mating container was a glass jar (15 cm long×3 cm diameter) covered with net (200 mesh nylon gauze). Each net had two holes the same size as tubes (1.5 cm diameter) (Fig. 2, B-2). Thus tubes could be inserted quickly (within 2–3 sec) into the mating jar (Fig. 2, A). Females and males could walk freely in the mating jar. Treatments were set up at the same time using different operators. Egg cards and cotton balls with 5% honey solution were introduced into containers before pairing (Fig. 2, B-1). The number of copulating moths was counted every 20 min for 10 h, and the start and end of each pairing was also recorded. In scotophase, matings were checked using a torch covered with red cotton.

### Survival and Reproduction

Adult survival was checked 2 h after they were stressed. Adults were considered dead if their body and appendage did not move after touching with a brush. Surviving adults were paired and allowed to oviposit in a glass jar (15 cm long×3 cm diameter), and held at 25°C, 60–70% RH and 15L: 9D. New cotton balls with 5% honey solution were supplied daily. Survivors and eggs on the wall of the glass jar and egg cards were counted daily until death. Egg cards were moved daily into clean tubes (10 cm length×1.5 cm diameter) and incubated at 25°C, 60–70% RH and 15L: 9D. The number of hatching eggs on the egg cards was recorded every day until all eggs hatched or collapsed. Male longevity data were inadvertently lost for the 3 h stress treatment. Temperature and humidity in the chamber were monitored by HOBO loggers (Pro V2 Temp/RH Data Logger U23-001, Onset Ltd., USA), and temperature variation was ±1°C. Details of the treatments are summarized in Table 1.

**Table 1:** Summary of traits scored in the experiments where females and/or males were exposed at 40°C for 0 h (Control), 3 h, 4 h or 5 h.

Exposure (h)	Copulation	Fecundity	Fertility	Female longevity	Male longevity
0 (Control)	Y	Y	Y	Y	Y
3	Y	Y	Y	Y	–
4	–	Y	Y	Y	Y
5	–	Y	Y	Y	Y

Y: data collected for the treatment; –: no data collected.

### Statistical Analysis

A contingency analysis was used to compare mating success of the four treatments in the 10 h period after pairing by computing the chi-square statistic. For successful matings, the time taken for 50% (CopT50) of the moths to copulate was determined by converting the cumulative percentage of mating success over 10 h to probit units and using a linear regression (y = a+bx, where x is the time after pairing in minutes and y is in probit units) to estimate time.

To examine the effects of treatment on copulation duration, longevity and reproductive traits, one-way ANOVAs (followed by Duncan’s post hoc comparisons) were run. In addition, to separate the effects of heat stress of the sexes on traits, two-way ANOVAs followed by Duncan’s post hoc tests were used with female and male temperature treatments as fixed factors. In these analyses, oviposition period was defined as the period of time required for 90% of total egg production. Fecundity was determined as the number of eggs laid on the wall of the glass jar and egg cards every day. Hatching success was determined as the percentage of eggs hatched, as computed from all eggs on the egg card. Fertility was evaluated as the fecundity multiplied by the egg hatching success. All statistical tests were performed using SAS V8 (SAS Institute, Cary, NC). The relationship of early fecundity in the first 2 days and pre-mating period after 3 h exposure was analyzed by fitting a polynomial line using the program Sigmaplot (SPSS Science).

## Results

### Survival and Longevity

Heat exposures for 3 h, 4 h or 5 h did not result in immediate mortality, which ranged from 0 to 1.1% in the treatments. Exposures for 4 h or 5 h also had no effects on female longevity (Fig. 3, A; 4 h: F_(3,82)_  = 1.78, P  = 0.157; 5 h: F_(3,74)_  = 0.86, P  = 0.468), with a mean (±SD) longevity across all treatments of 12.6±4.0 d. Females stressed at 40°C for 3 h lived 5.1 d longer than controls (both sex at 25°C) when mated with unstressed males (Fig. 3, A; 3 h: F_(3,74)_  = 3.31, P  = 0.025). Male longevity was not affected by heat treatments (Fig. 3, B; 4 h: F_(3,76)_  = 0.63, P  = 0.595; 5 h: F_(3,70)_  = 2.20, P  = 0.096), with a mean (±SD) longevity of 28.3±9.7 d.

### Mating Traits

Adults stressed at noon successfully mated by the night of the same day, with 87.7% of adults mating. The stress period may have reduced mating success somewhat, although the difference between the four treatments was not significant (*X* ^2^_(3)_  = 7.43, P  = 0.059). When only the female or male had been stressed, mating success tended to be lower (86.4% or 73.9% respectively) than when neither sex had been stressed (100% mating success) or when both sexes had been stressed (90.5% mating success). For successful matings, the 3 h heat shock treatment also did not affect copulation duration (F_(3,77)_  = 1.71, P  = 0.172), with an overall mean (±SD) copulation period of 88±27 min.

**Figure 4:**
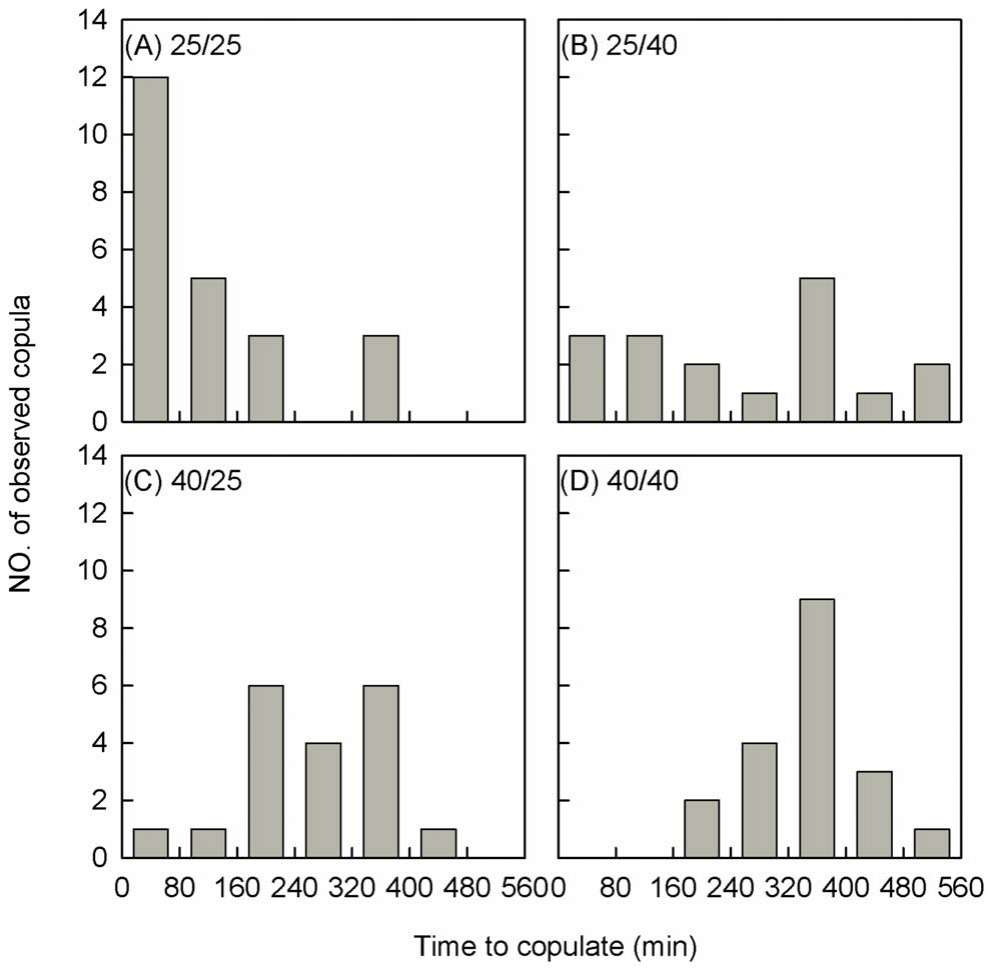
Number of copula observed in the 10 There were four treatments involving combinations of sexes held at 25°C (control) or stressed at 40°C for 3 h. This led to female/male treatments (A) 25/25, (B) 25/40, (C) 40/25 and (D) 40/40.

While any effects of the stress on mating success were minor, the distribution of copulations across time was affected (Fig. 4, A-D), with mating being delayed by heat stress imposed on males, females or both sexes. For the controls, the copulation peak (52% of total matings) occurred within 80 min after release (Fig. 4, A). With heat stress imposed only on males, matings proceeded evenly across time (CopT50 = 214 min), with 29% of matings occurring late within the 320–400 min interval (Fig. 4, B). When only females were stressed, copulation was delayed (CopT50 = 240 min), and 84% of matings occurred between 160–400 min after paring (Fig. 4, C). When both sexes were stressed, matings were further delayed (CopT50 = 317 min), and 84% of matings occurred between 240–480 min (Fig. 4, D).

### Reproduction

**Figure 5:**
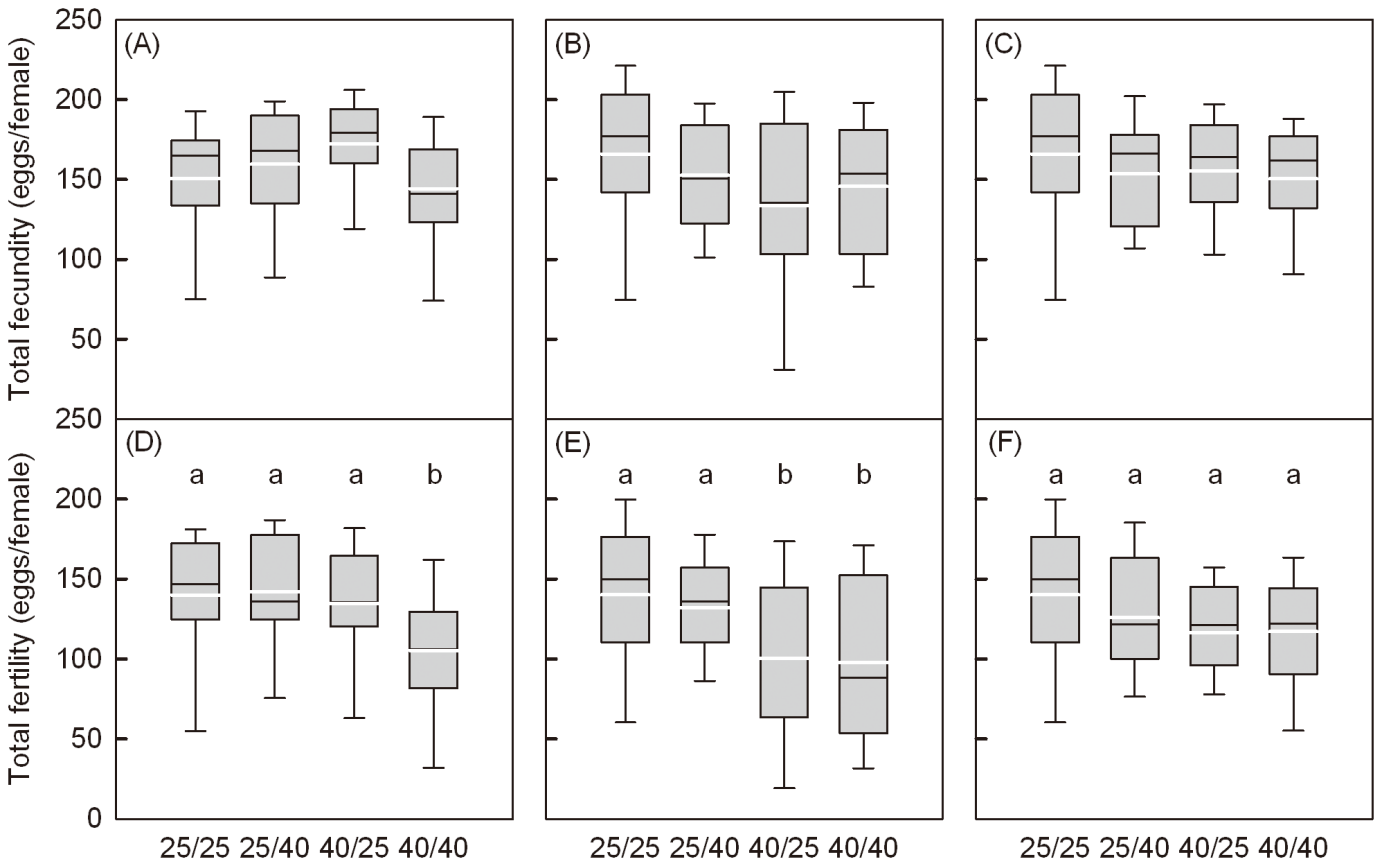
Box plots for total fecundity and fertility after adults heat stress. (A–C) refers to total fecundity and (D-E) to fertility per female. Treatments involved combinations of females and males exposed to control (25°C) or stress (40°C) for different exposure times of 3 h (A, D), 4 h (B, E) or 5 h (C, F). Different lowercase letters indicate significant differences between four treatment groups.

Females produced a mean (±SD) of 152±42 eggs. Females that had been stressed had a similar fecundity than the controls (both sexes at 25°C) (Fig. 5, A-C) and treatment differences were not significant (3 h: F_(3,73)_  = 1.85, P  = 0.146; 4 h: F_(3,79)_  = 1.64, P  = 0.188; 5 h: F_(3,70)_  = 0.48, P  = 0.694). However, the total number of hatched eggs was significantly reduced when females and/or males were exposed to 40°C for 3 or 4 h relative to the controls (Fig. 5, D-F; 3 h: F_(3,73)_  = 3.27, P  = 0.026; 4 h: F_(3,79)_  = 4.01, P  = 0.011; 5 h: F_(3,70)_  = 1.34, P  = 0.268).

**Figure 6:**
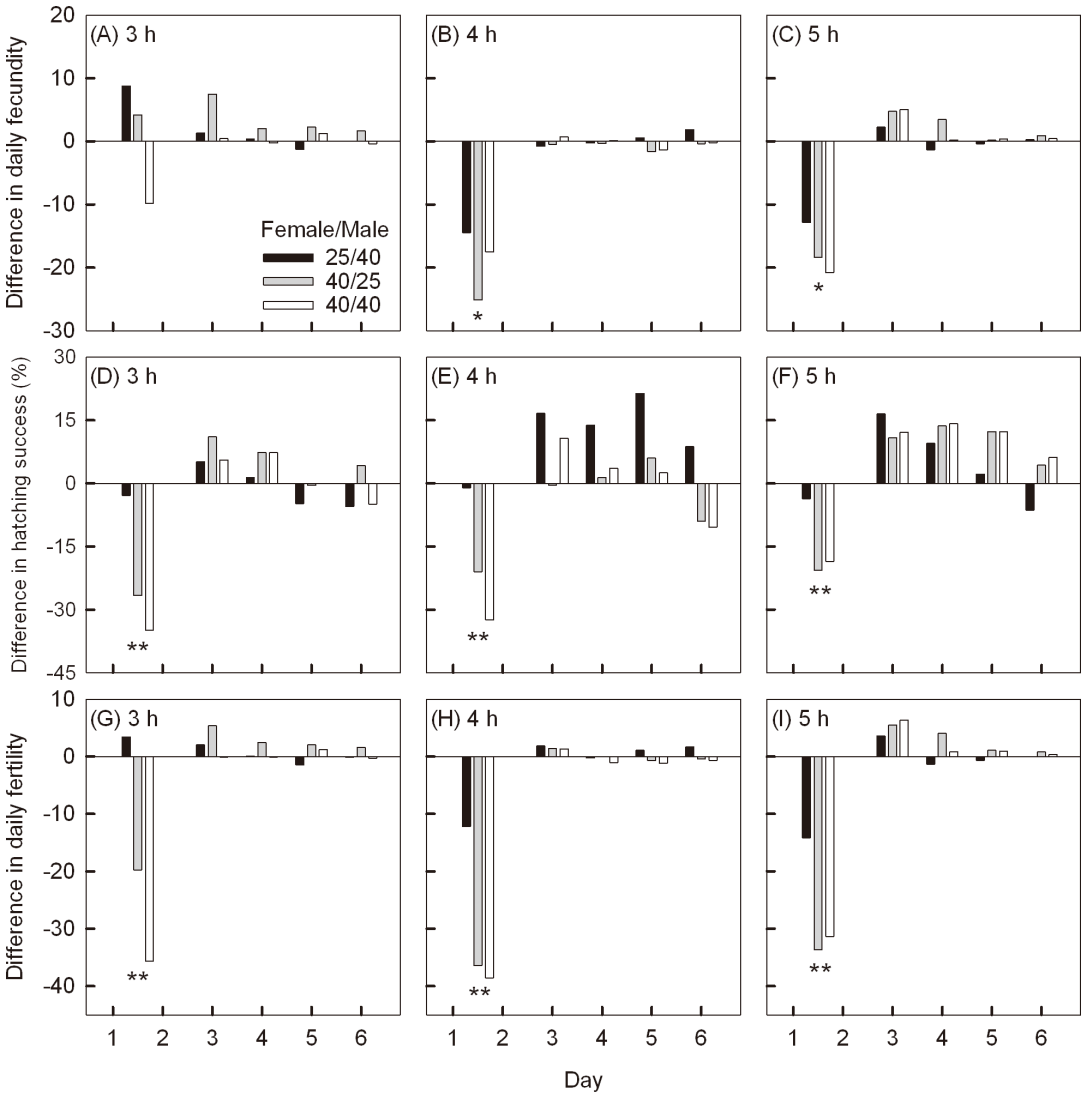
Effects of treatments on daily reproductive traits for the first 6 days. Bars present the difference of mean daily fecundity (A–C), daily egg hatching success (C–F), and daily egg fertility (G–I) of treated groups from the control (both sexes at 25°C) and after heat treatment for 3 (A, C and G), 4 (B, E and H) or 5 h (C, F and I). The asterisks below each plot indicate significant differences between the four treatments (*P<0.05, **P<0.01).

To further examine the influence of heat exposures, we considered daily egg production (Fig. 6, A, B and C), egg hatching success (Fig. 6, D, E and F) and egg fertility (Fig. 6, G, H and I) across 6 days of oviposition. Heat stress had no effect on the length of the oviposition period (3 h: F_(3,73)_  = 2.32, P  = 0.083; 4 h: F_(3,79)_  = 1.55, P  = 0.209; 5 h: F_(3,70)_  = 0.39, P  = 0.760), with females laying 90% of their eggs on average over 5.4±1.5 days. But when either females or males were exposed to 40°C for either 4 h or 5 h before mating, females tended to lay fewer eggs in the first 2 days when compared to the controls (Fig. 6, A-C, 3 h: F_(3,73)_  = 2.23, P  = 0.092; 4 h: F_(3,79)_  = 3.26, P  = 0.026; 5 h: F_(3,70)_  = 3.46, P  = 0.021). Heat stress also reduced the hatching success of eggs on the first 2 days (Fig. 6, D-F; 3 h: F_(3,73)_  = 15.07, P<0.0001; 4 h: F_(3,79)_  = 12.18, P<0.0001; 5 h: F_(3,70)_  = 6.69, P  = 0.0005), resulting in a significant decrease in the number of hatched eggs over this period (Fig. 6, G-I; 3 h: F_(3,73)_  = 9.68, P<0.0001; 4 h: F_(3,79)_  = 8.59, P<0.0001; 5 h: F_(3,70)_  = 7.66, P = 0.0002). However, these negative effects were no longer evident on the 3^rd^ day (Fig. 6) when differences between treatments and controls were non-significant (P>0.09) for all comparisons involving egg and egg fertility. Two days after moths had been transferred back to normal temperatures, moths that had been stressed tended to have a higher egg hatching success than the controls (both sexes at 25°C), although treatment effects were not significant (P>0.280).

To separate the influence of heat exposures on females from those on males, we considered daily egg production (Fig. 6, A, B and C), hatching success (Fig. 6, D, E and F) and egg fertility (Fig. 6, G, H and I) on the first two days. Females exposed to 40°C for 4 h or 5 h exhibited a larger reduction in egg production than when males exposed to these stresses (Fig. 6, A-C; 4 h: F_(1,79)_  = 6.01, P  = 0.017; 5 h: F_(1,70)_  = 6.87, P  = 0.011). Stressed females produced significantly fewer hatched eggs when exposed to stressful conditions (Fig. 6, G-I; 3 h: F_(1,73)_  = 25.21, P<0.0001, 4 h: F_(1,79)_  = 24.55, P<0.0001; 5 h: F_(1,70)_  = 19.36, P<0.0001), reflecting a lower egg hatching rate (Fig. 6, D-F; 3 h: F_(1,73)_  = 41.98, P<0.0001, 4 h: F_(1,79)_  = 33.79, P<0.0001; 5 h: F_(1,70)_  = 19.27, P<0.0001).

## Discussion

This study considered the impacts of a single extreme hot day on the performance of a widespread pest of *Brassica oleracea* and other crops; a single stress period of 40°C was used because this stress occurs in cropping areas in China and also in other parts of the world. Our results suggest that while there are no lethal effects of this stress on DBM adult survival and no negative effects on mating success, total fecundity and longevity, the stressed DBM adults did experience a delay in mating, reduced early egg production and reduced hatching success. Overall stressed females produced fewer hatched eggs (up to 21% reduction for 3 or 4 h exposure) compared to unstressed females. It is not clear how these effects will translate into the performance of DBM populations under field conditions, but they could lead to increased susceptibility to predation and reduced reproductive output.

While mating behavior was transiently inhibited after heat stress, almost all moths mated within 10 h after pairing. Few studies consider the effect of stressful events on adult mating. Females use sex pheromones to stimulate male courtship behavior and provide specific information of their location. High temperatures might lead to a reduction of pheromone titer [39], [40], and may also disturb pheromone components and thereby affect mating success as in *Spodoptera exigua* [41]. For males, courtship displays by males can decline during recovery from heat shock [20], [42]. Thus mating is potentially influenced by hot events in different ways.

In our study, imposing heat stress on females or males to 40°C before mating reduced egg production and egg hatching success in the first 2 days (Fig. 6). These negative effects were no longer evident in the following days. Because the reduction of early fecundity in the first 2 days was not associated with delayed mating after heat treatment (R^2^ = 0.013, see Figure S1), any decline in early reproduction is likely to be attributable directly to high temperature exposure. In contrast to patterns for stressed *H. armigera* [12], *D. melanogaster* [11], [15], and *Bicyclus anynana* [43], we found effects on fertility immediately after heat exposure rather than after a few days. Heat exposure in females and males has previously been shown to strongly affect the reproductive fitness of various insect species [15], [44]–[49]. Some studies have found that heat stress leads to sterilized eggs through effects on male spermatogenesis [46], which may be more thermosensitive than oogenesis [50]. However, the absence of strong male effects following heat stress in DBM may reflect the fact that large numbers of sperm are transferred to the female in the first mating to fertilize all oocytes [37]. In contrast to the present results, few studies have found that female reproduction is sensitive to heat stress [43]. High temperatures may disturb neurohormonal regulation to reproduction by delayed egg maturation and yolk production [51]–[53]. Heat shock response usually results in a concomitant reduction in the synthesis of yolk nutrition and chorion component [54] and the shortage of yolk nutrition may limit development even though eggs are incubated in an unstressed condition. Hsp70 production commonly induced after heat stress can be directly detrimental to egg hatching [55].

High temperatures result in a decrease of populations of DBM in summer in China, Japan and other countries. Predictions of the population dynamics of DBM are usually based on daily mean temperature [56]–[58]. However the current results suggest that high temperatures for only a short period influence fitness. Hatching rate seems particularly sensitive in comparison to survival and egg production of adults, pointing to maternal effects that could be incorporated into population dynamic models to improve prediction.

Finally, the results of this study point to practical ways in which pest control strategies might be implemented during hot conditions. DBM mating starts at the onset of darkness and peaking within an hour [38], [59]. This information has been used to control DBM by applying sprinkler irrigation to brassica crops at dusk to disrupt the insect mating [29]. However, the current results suggest that temperature should also be considered when determining the timing of irrigation, given that stressed DBM adults delay mating for 4–7 h. Irrigation might then be initiated later when high afternoon temperatures are experienced.

## Supporting Information

Figure S1 **The relationship of the early fecundity and pre-mating period after heat exposure.** The linear regression analysis showed that delayed mating after heat exposures for 3 h had no significant effect on the early egg production in the first two days (R^2^ = 0.013).
(TIF)
