# Supplementary figures and images for: A Single Hot Event That Does Not Affect Survival but Decreases Reproduction in the Diamondback Moth, Plutella xylostella
Source: PLoS One. 2013 Oct 8;8(10):e75923. doi: 10.1371/journal.pone.0075923 (PMC3793006; doi:10.1371/journal.pone.0075923)

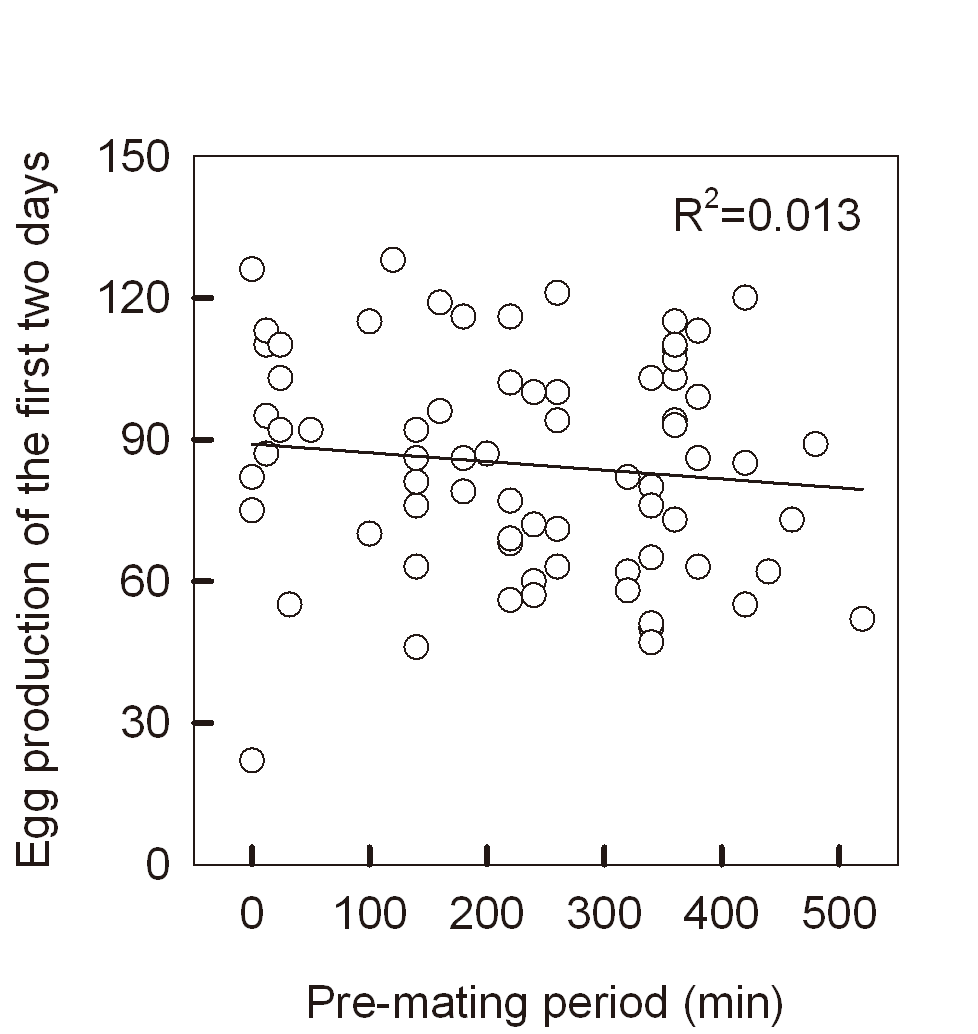

Supplement: Figure S1 — The relationship of the early fecundity and pre-mating period after heat exposure. The linear regression analysis showed that delayed mating after heat exposures for 3 h had no significant effect on the early egg production in the first two days (R2 = 0.013). (TIF) [file pone.0075923.s001.tif]
